# Psychological distress and associated factors among the attendees of traditional healing practices in Jinja and Iganga districts, Eastern Uganda: a cross-sectional study

**DOI:** 10.1186/1752-4458-2-16

**Published:** 2008-12-23

**Authors:** Catherine Abbo, Solvig Ekblad, Paul Waako, Elialilia Okello, Wilson Muhwezi, Seggane Musisi

**Affiliations:** 1 Karolinska Institutet, Department of Clinical Neuroscience, Section of Psychiatry, Stockholm, Sweden; 2 Department of Psychiatry, College of Health Sciences, Makerere University, P.O box 7072, Kampala, Uganda; 3 Stress Research Institute, Stockholm University, Stockholm, Sweden; 4 Department of Pharmacology and Therapeutics, College of Health Sciences, Makerere University, BOX 7072, Kampala, Uganda

## Abstract

**Background:**

Mental health problems are a major public health concern worldwide. Evidence shows that African communities, including Uganda, use both modern and traditional healing systems. There is limited literature about the magnitude of psychological distress and associated factors among attendees of traditional healing practices. This study aimed to determine the prevalence and associated factors of psychological distress among attendees of traditional healing practices in two districts in Uganda.

**Methods:**

Face-to-face interviews with the Lusoga version of the Self Reporting Questionnaire (SRQ-20) were carried out with 400 patients over the age of 18 years attending traditional healing in Iganga and Jinja districts in Eastern Uganda. Patients were recruited consecutively in all the traditional healers' shrines that could be visited in the area. Persons with 6 or more positive responses to the SRQ were identified as having psychological distress. Prevalence was estimated and odds ratios of having psychological distress were obtained with multiple logistic regression analysis.

**Results:**

387 questionnaire responses were analyzed. The prevalence of psychological distress in connection with attendance at the traditional healers' shrines was 65.1%. Having a co-wife and having more than four children were significantly associated with psyclogical distress. Among the socioeconomic indicators, lack of food and having debts were significantly associated with psychological distress. The distressed group was more likely to need explanations for ill health. Those who visited both the healer and a health unit were less likely to be distressed.

**Conclusion:**

This study provides evidence that a substantial proportion of attendees of traditional healing practices suffer from psychological distress. Associated factors include poverty, number of children, polygamy, reason for visiting the healer and use of both traditional healing and biomedical health units. These findings may be useful for policy makers and biomedical health workers for the engagement with traditional healers.

## Background

The World Health Organisation (WHO) states that the quarter of the world's population who have common forms of mental illness should be treated in Primary Health Care (PHC) settings[1]. However, research from many African countries suggests that the WHO's plea for PHC workers to deliver mental health services is yet to be heeded [2-5]. There may be a number of reasons for this but the most notable are the burden of infectious diseases in PHC settings in Africa and/or lack of understanding of patients' explanatory models of mental illness among providers of PHC services [4,6-8]. According to the WHO, traditional healers appear to be an important entry point on the pathway to care for people who ultimately use psychiatric services [9].

One of WHO's goals is to promote appropriate mental health policies for maximum utilization of locally available resources at country level, including traditional healers' services. In Uganda, the Ministry of Health's policy regarding traditional medicine has been to call for its recognition and incorporation in the country's health care delivery [10]. However, no legal framework is in place for the practice of traditional medicine. This lack of a legal framework and mechanisms for implementation has made it difficult to implement the policy[11].

Traditional healing existed in Africa long before the introduction of biomedicine. With the advent of biomedicine, formal health service systems tended to reject traditional healing. In many cases, the practice was even prohibited [12].

Indeed, the only law about traditional practices that still exists in Uganda is the 1964 Witchcraft Act, which stipulates penalties against intended acts of harm [13]. Notwithstanding its many successes and general acceptance, biomedical practice has not replaced traditional healing; instead, they exist side-by-side [12,14]. This may be because traditional practice is deeply embedded in the wider belief and cultural systems and remains an integral part of the lives of most Ugandans.

The available information in Uganda indicates that a majority of users of traditional healers' services have psychological and social problems [14]. These problems are often work-related issues, for example bad lack in getting a job, no success at a job, marital problems, problems with neighbours, politics and family. When distressed or ill, people often turn for help to family members or others around them. Those people may give advice and suggest where to go for help if the problem continues. Popular perceptions of what is appropriate care usually influence the advice, as do cultural and religious factors as well as previous experiences with healing (positive or negative) [15].

There is no systematic documentation of either the prevalence of psychological distress among persons attending traditional healing practices or of the socio-economic characteristics of people with psychological distress who use these services in Uganda. In this study, psychological distress is defined as the experience of disruption in meaning, understanding, and smooth functioning, with possible consequences for the person in the form of harm, loss or challenge [16], and with both physical and psychological consequences [17]. Mental health and mental illness are not polar opposites, but points on a continuum. Somewhere in the middle of that continuum are "mental health problems," which most people have experienced at some point in their lives. A familiar example is the experience of feeling low and dispirited in the face of a stressful job. Mental health problems may translate into psychological distress, depending on the number of symptoms present. The boundaries between mental health problems and milder forms of mental illness are often indistinct, just as they are in many other areas of health. Yet at the far end of the continuum lie disabling mental illnesses such as major depression, schizophrenia, and bipolar disorder. Left untreated, these disorders have a devastating potential. Not everyone with psychological distress has mental illness. However, people with mental illness are unlikely not to be psychologically distressed. This study measured psychological distress regardless of whether or not it was accompanied by distinct mental illness.

Findings about the relationship between socio-economic characteristics and the risk of psychological distress are somewhat contradictory. The study by Shekhar et al [18] indicates that the incidence of mental disorder and the need for care are highest among poor people in rural communities. Furthermore, studies in Ethiopia and other parts of Africa have indicated that risks of psychological distress are highest among the poorest, the least educated, and the unemployed [19,20]. On the other hand, a study in Tanzania found that neither gender, age, education nor any other indicators of socio-economic status were significantly associated with psychological distress [21].

The aim of this study was to determine the prevalence of psychological distress, as well as its associations with socio-demographic characteristics, among persons attending traditional healing practices in two districts in Uganda. The evaluation also included the association between psychological distress and reasons for visiting the traditional healer, the kinds of treatment sought and the duration of illness among persons attending traditional healing practices in Eastern Uganda.

## Methods

### The study setting

This study was conducted in the Busoga region of Eastern Uganda. Busoga is a kingdom comprising the 11 principalities of the Basoga people, one of the largest of the five traditional kingdoms in present-day Uganda. The kingdom's capital is located in Bugembe, which is near Jinja, the second largest city in Uganda. The three million Basoga (*2002 census*) make up the second largest (*after the Baganda*) Ugandan ethnic group or tribe, although they represent only about 11 percent of the population. The Basoga are located between two large ethnic groups: the Bantu to the South and West and the Luo to the North and East. This study was carried out in two of the districts in the Busoga region: Iganga and Jinja.

Of the 240 registered traditional healers in the two districts, 150 (62.5%) were visited. All these 150 healers had a place of practice. For most of them, this was separate from their place of abode but nearby or within the compound. This place of practice or shrine was often composed of grass-thatched mud huts, with the floor covered with raffia mats (*mikeeka*). Those close to town had been modernized by cementing the walls and floor.

At the time of the visit, the number of patients at the traditional healer's shrine ranged from 1–5 attendees per day.

Some clients were in-patients, hospitalized in the huts. In all the shrines, one corner usually had containers for medicines in the form of dried leaves, roots, tree bark of varying texture, animal skins, dried animal meat or bark cloth.

The traditional healer sat on bark cloth or an animal skin. In front of the healer were usually the 'diagnostic' tools, which included cowrie shells, beads or sticks wrapped in bark cloth and placed in a basket. A few traditional healers had the *'Quaran' *(the Muslim prayer-book) as a tool for diagnosis and solutions. None had the *Bible*. This could be because Islamic religion in Africa incorporated African tradition, while Christianity rejected and continues to be antagonistic to traditional healings [14].

Usually, a curtain of bark cloth separated the sleeping or resting area from the working area. There were no beds. Clients who stayed overnight slept on the floor. The shrines either had a fire burning continuously in one corner or a charcoal stove burning most of the time in the same room. No modern furniture would be allowed in the shrines, only the traditional raffia of mats (*mikeeka*), bark cloth, dry grass or animal skins.

### Study Design

This was a cross-sectional study conducted to determine the prevalence of psychological distress and associated factors among attendees of traditional healing practices in Jinja and Iganga districts of Uganda between January and March 2008.

### Sample size and selection

Sample size was estimated with the Kish Leslie [22] formula for single proportions for descriptive study. The estimate started from the assumption of a 48% prevalence of psychological distress at the traditional healer's facility [21,23]. Together with a 95% confidence interval with a 5% level of precision, this led to the consecutive recruitment of a total of 400 clients. The level of significance was set at p < 0.05.

The study concentrated on registered traditional healers practicing in the two districts. This is because these healers are considered to be genuine, while quacks are less likely to be registered. Traditional healers are scattered in the villages; research assistants (Psychiatric Assistants) interviewed 150 of the registered traditional healers who could be reached despite adverse terrain, a poor road network and a lack of reliable means of transport. The number of patients per traditional healer per district was worked out proportionately. This gave the following figures: a total of 300 patients were recruited in Jinja district and a total of 100 patients in Iganga district, making a grand total of 400 patients.

### Study Subjects

The study subjects were patients aged 18 years or more who were attending the traditional healers' shrines for some ailment in the study period. These patients or their caregivers voluntarily consented to the study.

### Measures

The study instruments were:

#### (a) A socio-demographic questionnaire

This consisted of prepared socio-demographic questions comprising items on age, gender, marital status, tribe, religious affiliation, educational attainment, number of children, gainful employment, household income in Uganda shillings, whether in debt or not and whether respondents had slept without food for lack of it at least once in the past month. Additional questions included: reason for visiting the shrine, kind of treatment given for the complaints and duration of symptoms.

#### b) The Self-Reporting Questionnaire (SRQ-20)

The Self-Reporting Questionnaire (SRQ-20) was used for measuring psychological distress in attendees of traditional healing practices. The SRQ-20 was derived from four psychiatric morbidity instruments from a wide variety of cultural backgrounds [24]. It was developed for a WHO collaborative study to screen for common mental disorders in primary health care. The SRQ-20 has been validated in Uganda and a cut-off point of 6 was taken to be indicative of psychological distress [25]. Other African studies have had similar cut-off points [20,24,26,27].

The SRQ -20 was then translated into Lusoga, the language of the study area, and back translated by an independent psychiatric assistant from the study area. The translated version of the SRQ-20 was pre-tested with a sample size of 40 (recommended 10% of study sample size) in a community sample that was similar to but outside the sampled districts.

The SRQ-20 is designed to be self-administered. In this study, however, the low literacy of the study population made administration by an interviewer more appropriate. The Lusoga version of SRQ was used to collect data. The interviewers read it to the respondents. Since the SRQ-20 is not a substitute for or equivalent to a clinical diagnosis, the interviewers read the questions once to the respondents and repeated them only if they were not clear; they did not have to elaborate by paraphrasing. The responses were 'Yes' or 'No' and referred to current existence of particular symptoms.

### Data collection

The trained and supervised interviewers we employed to collect data were Psychiatric Assistants. In Uganda, a psychiatric assistant is a health provider trained to the level of a registered nurse and later given two more years of training in diagnosis and treatment of mental disorders. Before data collection began, the district officials and the traditional healers were officially contacted to inform them about the purposes of the study and to ask for permission. Traditional healers were also asked to assign someone to serve as a guide to the interviewers. A standard set of procedures and statements was prepared for the interviewers to introduce themselves to the traditional healers, especially those they had not met before. The traditional healers were asked for permission to talk to their clients and the clients were asked to voluntarily consent to participate. Refusal to participate was minimal. Six traditional healers (0.015%) refused to let their patients be interviewed. However, this was unlikely to have affected the results because other traditional healers within the same location were picked to replace them. No patient refused to participate in the study. If a traditional healer refused or no patients were present at the time, the next shrine was visited. There was at least one shrine in every village. In order to avoid visiting a sub-county twice, each research assistant and a local guide collected data in different sub-counties. In the case of a need for advice or referral, the trained psychiatric assistants gave the advice or made the referral after agreeing with the healer in charge.

### Ethical considerations

Ethical clearances were obtained from the following sources: the Human Research and Ethics Committee of Karolinska Institutet in Sweden (Dnr 05/07); the Research and Ethics Committees of Makerere University Medical School (Uganda); the Uganda National Council for Science and Technology Committee on study of Human Subjects (HS 323); the District Directors of the Health Services in the districts concerned; and, finally, the leaders of the traditional healer association in the two districts. Conduct during the study adhered to the Helsinki Declaration [28]. Participants in need of lifesaving attention were identified and, in consultation with the attending traditional healer, were offered emergency treatment, e.g. a patient having seizures would be given diazepam and referred to a health unit.

### Data management and statistical analysis

Completed forms were checked for completeness, consistency and accuracy on a daily basis and before data entry into a computer. For evaluation of associations between socio-demographic factors and psychological distress we used an SRQ cut-off point of at least 6 out of 20 items. This selection was based on reports from validation studies conducted in Uganda and elsewhere in Africa [20,24,26,27].

We used Epidata for data entry and the data analysis was performed using SPSS version 15.0 for Windows. Cronbach's α for internal consistency was used to test reliability of the SRQ 20 and was estimated at 0.85 for the SRQ scale. This was above the generally accepted minimum threshold of ≥0.70 for internal reliability coefficient[29].

Multivariate logistic regression was applied to produce odds ratios (OR) of associations between independent demographic variables and outcomes of cut-off points of SRQ, and adjusted to address the influence of other significant variables. Based upon the cut-off levels, the outcome of psychological distress was dichotomized into respondents exhibiting psychological distress (an SRQ score of ≥ 6 'yes' answers) and those not exhibiting psychological distress (an SRQ score of ≤ 5 'yes' answers).

Continuous independent variables were categorized for the analysis. All demographic data which were statistically significant ((p < 0.05) following a univariate analysis to test for the strength of association were included for the multivariate analysis.

The associations in the multivariate analysis that were statistically significant (p < 0.05), using a backward elimination regression approach, were included in the final results.

## Results

Of the 400 interviews, 13 (3.3%) were discarded because of incompleteness. The remaining 387 interviews (96.7%) were analyzed. The numbers of males and females were much the same (46% v 54.0%): Table 1 below summarizes these socio-demographics characteristics.

**Table 1:** Demographic characteristics of attendees of traditional healing practice (N = 387)

**Variables**	**Number (%)**
**Sex**	
Male	178 (46.0)
Female	209 (54.0)
**Age**	
18–24	101 (26.1)
25–34	108 (27.9)
35–44	88 (22.7)
45 and above	90 (23.3)
**Education**	
None	74 (19.1)
Some education	313 (80.9)
Secondary upwards	114 (29.5)
Primary (p1-p7)	199 (51.4)
**Occupation**	
Gainful (self, formal, casual)	164 (42.4)
Not gainful (Housewife, student, peasant)	223 (57.6)
**Household income**	
Less than $1 a day	350 (90.4)
More than $1 day	37(9.6)
**Lack of food in the past month**	
Slept hungry at least once	145 (37.5)
Did not sleep hungry	242 (62.5)
**Debts**	
In debt	256 (66.1)
Not in debt	131 (33.9)
**Marital status**	
Married	196 (50.6)
Unmarried	191 (49.4)
**Married males (N = 70)**	
Only 1 wife	41 (10.6)
≥1 wife	29 (7.5)
**Married females (N = 127)**	
The only wife	90 (23.3)
≥1 or more co wives	37 (9.6)
**Those who had children (N = 263)**	
1–4	133(34.4)
> 4	130(33.4)
**Tribe**	
Soga	336 (86.8)
Ganda	28 (7.2)
Others	23 (6.0)
**Religion**	
Christian (catholic, pentacostal, protestants)	239 (61.8)
Non Christian (Muslims, Traditional, others)	148 (38.2)
**Reasons for visiting healer**	
To get treatment	174 (45.0)
For treatment and explanation	213 (55.0)
**Kind of treatment received for current symptoms**	
Traditional	194 (50.1)
Traditional and modern	193 (49.9)
**Duration of symptoms**	
Less than six months	217 (56.1)
More than six months	171 (44.2)

A majority of the respondents, 297 (76.7%), were below 45 years of age; the mean age was 34.8 years (s.d. 13.55). Two hundred and twenty three (57.6%) were not in gainful employment and the majority 350 (90.4%) earned an equivalent of less than 1 dollar a day. One hundred and forty five (37.5%) had lacked food at least once in the past month and 256 (66.1%) were in debt. The respondents were mainly Basoga (336, 86.6%) and the majority, 313 (80.9%), had attained some education (primary 51.4%, secondary 29.5%). Christianity 239 (61.8%) was the predominant religion. The numbers of married and unmarried respondents were almost the same (50.6% and 49.4%).

### Prevalence of psychological distress

Of those attending traditional healing practices, 252 (65.1%) scored 6 or more on SRQ 20. They were therefore classified as having psychological distress. In terms of gender, 70.4% of the males were distressed compared to 61.2% of females but the difference is not statistically significant (*p *= 0.08, OR of 1.45 [95%CI 0.93–2.27].

### Relationship between socio-demographics and psychological distress

Table 2 presents the statistically significant (*p *< 0.05) adjusted odds ratio results of the multivariate logistic regression analysis of the associations between demographic variables and the outcomes of psychological distress. These show that married females with co-wives were over three times more likely to be distressed than those who were the only wife (*p *= 0.012; OR 3.62 [95%CI 1.38–10.98]). In contrast, married men with more than one wife seemed to be protected for psychological distress (*p *= 0.001; OR 0.20 [95%CI 0.06–0.63]).

**Table 2:** Relationship between some socio-demographic variables and psychological distress of the respondent

**Variable**	**Distressed** **(%)**	**Not distressed** **(%)**	**P value**	**OR (95%CI)**
**Married men (N = 70)**				
More than 1 wife	23.0	78.0	0.001	0.20 [0.06–0.63]
**Married women (N = 126)**				
More than 1 co-wife	68.8	31.2	0.012	3.65 [1.38–10.98]
**No of children (N = 263)**				
> 4	76.9	23.1	< 0.001	3.00 [1.71–5.29]
**Socioeconomic indicators**				
Lack of food	90.3	9.7	0.001	2.25 [1.25–4.72]
Being in debt	84.3	15.7	0.002	2.52 [1.40–4.53]
**Reasons for visiting T/H**				
To get treatment and explanation	73.7	26.3	0.02	2.17 [1.33–3.34]
**Kind of treatment**				
Both traditional and modern	42.0	58.0	<0.001	0.28 [0.18–0.44]
**Duration of symptoms**				
More than six months	69.6	30.4	0.032	1.65 [1.04–2.62]

Among other socio-economic indicators, persons who had lacked food at least once in the past month or who were in debt were twice as likely to be distressed; (*p *= 0.001; OR 2.52 [95% CI 1.25–4.72]) and (*p *= 0.002; OR 2.52 [95% CI 1.40–4.53]), respectively. Respondents who were attending the traditional healer's place both for treatment and for explanations for their illness were twice as more likely to be distressed (*p *= 0.02; OR 2.17 [95%CI 1.33–3.34]) as those who wanted treatment only. On the other hand, those who stated they had visited a health centre as well as the traditional healer for the same problems were less likely to be distressed (*p *< 0.001; OR 0.28 [95%CI 0.18–0.44]).

## Discussion

The literature regarding the prevalence of psychological distress among people attending traditional healing practices in Africa is limited. In this study, the prevalence was 65.1%, which is high compared to 52% in a study by Okello et al in 2006 in four districts of Uganda[14] and 48% by Ngoma et al in Tanzania [21]. In 1997, Patel et al reported a prevalence of 40% in Zimbabwe [19]. Our prevalence is twice as high as the prevalence of psychological distress in primary health care settings, which ranges from 10 to 30% [30-32]. These figures indicate that a majority of the patients who attend healers have psychological distress. In Africa, traditional healers are found within the community and are therefore more accessible than biomedical health workers [14]. In rural Uganda, the ratio of doctors to the population is about 1:30,000, whereas that of traditional healers is 1:100 [33].

### Risk factors for psychological distress among attendees of traditional healing

The association between socio-demographic factors and psychological distress has been demonstrated in previous studies [14]. In this study, the married women who had a co-wife were associated with more psychological distress than those without a co-wife. Among the married men, having more than one wife was not associated with psychological distress. We do not have a clear explanation for this. It could be that women in polygamous relationships are in a more stressful marital arrangement than those in monogamous relationships. In a Turkish study of women's mental health, a comparison of women from polygamous and monogamous families, respectively, found that the former showed more psychological distress than the latter [34].

Other possible explanations include less access to resources, reduced level of support, possible weak marital bonds, violence and jealousy [35]. All these factors may contribute to psychological distress in women in polygamous relationships. Further research is needed in this area to determine the associated factors.

In this study, we also found that having more than four children was significantly associated with psychological distress. In traditional Africa, having many children has always been considered an asset for a variety of reasons; for example, children would do farm work and other domestic chores. Today, however, children have to attend school; parents have to afford educational materials, school uniforms, meals and other educational needs, medical care, etc. All this makes raising children more of a burden than it used to be. In 2000 the Government of Uganda introduced free primary education for not more than four children from the same family. Parents are still supposed to meet the rest of their children's needs, including educational materials and meals.

This study found that some indicators of socio-economic status (being in debt, sleeping hungry because of lack of food) were significantly associated with psychological distress. The majority of the respondents were peasant farmers who were likely to have less education, less likely to be in gainful employment and earning less than one dollar a day. All these factors are related to poverty. The relationship between poverty and psychological distress, especially depression, is receiving increasing attention by researchers these days. Although no direct causal relationship has been demonstrated, it is widely recognized that extreme poverty causes much distress [36,37]. A study in northern Uganda found that the absence of basic social goods and services, such as food and clothing, had a significant association with outcomes of distress [37].

Another study by Ovuga et al in 2005 in the same region as this study found no association between depression, depressed mood and unemployment [38]. However, the majority of Ovuga's respondents were peasant farmers who, although they had no formal employment, were engaged in meaningful activities to sustain their lives and families. Our study suggests that this is no longer the case, as our respondents lacked food and were in debt, which points to both material and monetary poverty. It is also likely that poverty and gender may influence help-seeking differently for those with and without psychological distress [21]. Other factors influencing health-seeking behaviour may include belief systems, accessibility, availability of the provider and flexibility of payment terms [12].

There were significant associations between going for both treatment and explanations for the illness, having visited the healer as well as a health worker and the duration of symptoms. The distressed group was more likely to visit the shrine for treatment and for seeking explanations regarding their ill health. Much research in medical anthropology has developed the idea of explanatory models, which may include accounts of causality, mechanisms or processes of illness, illness course, appropriate treatment, expected outcome and consequences. Not all this knowledge is directly related to personal experience. Much of it resides in cultural practices transmitted by other people over generations. Hence, understanding the cultural meanings of symptoms and behaviour often requires interviews with other people in the patients' family, entourage or community [39]. In our study, we interviewed the patients about why they went to see the traditional healer but not about the types of explanation they received from the healer.

Those who visited the traditional healer and biomedical health units for the same problems were less likely to be distressed. Again, the mechanism here is not clear since psychological distress is usually not diagnosed at PHC [5,14,40]. Literature indicates that biomedical health workers focus more on physical illness than psychosocial problems, whereas traditional healers pay more attention to interpersonal and other psychosocial problems that cause psychological distress [5,7,40]. This may explain why people who used both systems seemed to have received more comprehensive care and hence a reduction of psychological distress. Also consultation in traditional healing practices is more likely to produce an illness identity which matches the patient's perceptions, thus making sense of the patient's real world [7,41,42]. However, our study is inconclusive regarding whether using of both traditional and modern treatments promotes better mental health. That calls for a study, which systemically charts the chronology of symptoms, pathways to care and treatments received.

## Study strengths and limitations

Traditional healers provide about 60–80% of health care needs in developing countries and this study is a contribution to the limited scientific research in this area. Secondly, all traditional healers who could be accessed were included in the study to avoid bias. Thirdly, although some studies have questioned the validity of SRQ-20 [43], this instrument has been used in Uganda and various other African countries and has been found to be feasible and valid for the assessment of psychological distress [25,43,44]. It has been validated in central Uganda, a culture similar to the culture studied here. It is easy to translate and therefore readily usable in African settings, including Uganda. In this study, the translated version of SRQ-20 was found to have high internal reliability levels as assessed by Cronbach's alpha.

The limitations of this study include the fact that we used a descriptive cross-sectional design and are therefore unable to draw conclusions about causal connections between socio-demographic factors and psychological distress. In the assessment of socioeconomic indicators, we only used lack of food in the past month and being in debt; other indicators of socio-economic status, such as type of housing, were not included. Finally, this study was conducted in only two districts of Eastern Uganda. Uganda being a multicultural society, the results may not be generalisable. Despite the inclusion of all accessible traditional healers, we cannot claim that the participants were a true representative sample of all those who attend traditional healing. Those who attend at night had to be left out because of the study's logistics. For example, these night patients may have different characteristics from those who visit during the day.

## Conclusion

The prevalence of psychological distress among persons attending traditional healing practices was high. This was the case regardless of sex, age, education, occupation, marital status, religion or tribe.

People with psychological distress make use of a range of healing systems, only one of which is traditional healing. It therefore seems logical to conclude that traditional healers make a contribution to the provision of mental health care services in Uganda. Consequently, efforts to improve the quality of mental health care services within the currently available resources will require biomedical mental health service providers to engage traditional healers so as to ensure that appropriate mental health is accessed by those who need it [15].

From this study, the combined use of traditional healers and modern health facilities seems to lessen psychological distress. Much work needs to be done which critically examines the interface between traditional healing and biomedical care, in order to gain the best from both approaches in the interest of those who use these services.

## Abbreviations

These include: PHC: (Primary Health Care); WHO: (World Health Organization); T/H: (Traditional Healer); MoH: (Ministry of Health); GoU: (Government of Uganda); WMA: (World Medical Association).

## Competing interests

The authors declare that they have no competing interests.

## Authors' contributions

All authors have participated in the study from its inception. Catherine Abbo supervised data collection; analysed data assisted by Wilson Muhwezi and wrote the manuscript. Solvig Ekblad, Elialilia Okello, Paul Waako and Seggane Musisi were the overall supervisors and reviewed the manuscript. All authors read and approved the manuscript.
